# Screening and Evaluation of tRF-Glu-CTC-013 as a Biomarker and Key Regulator in the Development of Cardiac Hypertrophy

**DOI:** 10.7150/ijms.106114

**Published:** 2025-06-09

**Authors:** Wenlin Li, Ming Lan, Kun Xu, Sainan Li, Que Wang, Beidong Chen, Xiuqing Huang, Lin Dou, Na Jia, Li Zhao, Yuefeng Wang, Xingyun Jiao, Yong Man, Deping Liu, Liang Sun, Tong Zou, Qing He, Jian Li, Xue Yu, Tao Shen

**Affiliations:** 1 The Key Laboratory of Geriatrics, Beijing Institute of Geriatrics, Institute of Geriatric Medicine, Chinese Academy of Medical Sciences, Beijing Hospital/National Center of Gerontology of National Health Commission, Beijing 100730, China.; 2 Department of Cardiology, Beijing Hospital, National Center of Gerontology, Institute of Geriatric Medicine, Chinese Academy of Medical Sciences & Peking Union Medical College, Beijing 100730, China.; 3 Graduate School of Peking Union Medical College, Beijing 100730, China.; 4 Department of Gastroenterology, Chinese Academy of Medical Sciences, Beijing Hospital/National Center of Gerontology of National Health Commission, Beijing 100730, China.

**Keywords:** cardiac hypertrophy, tRNA-derived stress-induced RNA (tiRNA), tRNA-derived fragment (tRF), tsRNA, ventricular remodeling

## Abstract

tRNA-derived small RNAs (tsRNAs) are a newly recognized class of non-coding RNAs involved in regulating RNA processing and translational control. Pathological cardiac hypertrophy, characterized by left ventricular remodeling under chronic stress, serves as a critical precursor to severe cardiovascular pathologies including myocardial ischemia, infarction, and heart failure. Utilizing an angiotensin II (Ang II)-induced mouse cardiac hypertrophy model combined with tsRNA transcriptome profiling, we identified differentially expressed tsRNAs and investigated their functional relevance. Validation in neonatal mouse ventricular myocytes (NMVMs) revealed five upregulated tsRNAs associated with hypertrophic progression. Functional characterization showed that overexpressing tRF-Glu-CTC-013 significantly reduced cardiomyocyte hypertrophy and inhibited inflammation and fibrosis. Further luciferase reporter assays revealed that tRF-Glu-CTC-013 could bind to the 3' UTR of TAS1R3, thereby inhibiting its expression and enhancing the level of autophagy in NMVMs. Taken together, these findings suggest that tsRNAs may act as novel regulators of cardiac remodeling, with tRF-Glu-CTC-013 emerging as a promising therapeutic candidate for cardioprotection via anti-hypertrophic, anti-inflammatory, and anti-fibrotic mechanisms.

## 1. Introduction

Cardiovascular diseases remain a leading cause of global morbidity and mortality, exacerbated by demographic aging and lifestyle-related risk factors^1^. Pathological cardiac hypertrophy is a compensatory response of the heart to adapt to its environment following various pathological stimuli such as hypertension, valvular disease and myocardial infarction^2^, ^3^. In the early stages of cardiovascular disease, the heart experiences increased stress, resulting in structural and functional changes characterized by cardiomyocyte damage, cardiac fibrosis, and cardiac dysfunction. These changes can lead to cardiac hypertrophy^4^. The compensatory phase is characterized by an increase in the volume of cardiomyocytes and the external matrix, and the decompensatory phase follows with a continuous decline in cardiac function and thinning of the ventricular wall, which may give rise to malignant arrhythmias, heart failure and even death^2^, ^3^, ^5^. This pathological progression is driven by complex molecular mechanisms involving oxidative stress, calcium dysregulation, mitochondrial impairment, and noncoding RNA networks^6^-^8^. In recent years, evidence from many studies has suggested that tsRNAs play a key regulatory role in these pathological processes.

Once considered mere tRNA degradation byproducts, tsRNAs have gained recognition as functional regulators through advances in deep sequencing and noncoding RNA biology^9^. The Dicer enzyme, ElaC ribonuclease Z2, angiopoietin and other RNases degrade tRNA to form tiRNA and tRF^10^, ^11^. tsRNAs function similarly to microRNAs (miRNAs) and also play a role in regulating gene expression and protein translation^10^. tsRNAs have been shown to play a role in the pathological process of cardiac hypertrophy, and can even contribute to its intergenerational inheritance^12^. Shen used an isoprenaline (ISO)-induced rat model of cardiac hypertrophy to compare the expression of tRFs in rat spermatozoa and offspring hearts between the cardiac hypertrophy group and the control group. tRF was enriched in hearts with cardiac hypertrophy. The overexpression of both tRFs1 and tRFs2 in H9c2 cells increased the surface area of cardiomyocytes and the expression of biomarkers of cardiac hypertrophy^13^. Luciferase reporter analysis revealed that tRFs1 derived from tRNA-Gly-GCC directly targets the 3'UTR of tissue inhibitor of metalloproteinases 3 (TIMP3) to inhibit its expression. Shen also reported that tRF is involved in regulating the response to cardiac hypertrophy, suggesting that it may act as a novel epigenetic factor promoting intergenerational inheritance of cardiac hypertrophy. Previous studies have shown that tsRNAs are also involved in the regulation of autophagy in cardiomyocytes. In cardiac progenitor cells treated with high glucose, tRF-5014a was found to negatively regulate the expression of the autophagy-related protein ATG5. Inhibiting tRF-5014a reversed the inhibition of autophagy, improved cell viability, and reduced the release of pro-inflammatory cytokines under high-glucose conditions^14^. Despite these advances, the role of tsRNAs in cardiac hypertrophy and the underlying molecular mechanisms remain largely unexplored.

Several clinical studies have identified tsRNAs as good biomarkers or potential therapeutic targets for cardiovascular disease. A study involving the sequencing of small RNAs in the plasma of healthy volunteers and patients with pathological cardiac hypertrophy identified plasma tRF-21-NB8PLML3E as a potential biomarker for the early screening of pathological cardiac hypertrophy. Furthermore, the tRF-21-NB8PLML3E mimic decreased the levels of ANP and BNP and reduced the surface area of Ang II-induced H9c2 cells^15^. tsRNA is also differentially expressed in the epicardial adipose tissue of patients with heart failure, and may influence the function of cardiomyocytes via exosomes^16^. Lu et al. reported that tsRNAs could be used to diagnose congenital heart disease in foetuses by sequencing peripheral blood samples taken from pregnant women^17^. In diabetic cardiomyopathy (DCM), tRFs may be responsible for high-glucose-induced myocardial injury^14^.

In this study, we investigated changes to tsRNAs in Ang II-induced cardiac hypertrophy. Through comprehensive transcriptomic and functional analyses, we identified tRF-Glu-CTC-013 as a key regulator of cardiac hypertrophy. It achieves this by reducing TAS1R3 expression and promoting autophagy function. Our findings improve our understanding of the mechanisms of tsRNA-mediated cardiac adaptation, suggesting new target genes and strategies for treating cardiac hypertrophy.

## 2. Materials and Methods

### 2.1. Animal experiments

Ten- to 12-week-old male C57BL/6J mice and one- to three-day-old neonatal C57BL/6J mice were purchased from SiPeiFu (Beijing) Biotechnology Co. Ltd., and were housed on specific pathogen-free platforms at a controlled temperature of 22-24 °C and humidity of 55-60%, with a 12-hour light-dark cycle. The mice had free access to food and water. Animal experiments were conducted in accordance with the Guide for the Care and Use of Laboratory Animals (published by the National Institutes of Health, 2011 revision) and were approved by the Animal Use and Care Committee of Peking University Health Centre. The mice were randomly divided into three groups: the control group (Con group), the Ang II group (A group) and the neonatal group (N group). Angiotensin II (Ang II) was purchased from EMD Millipore Corp. (Cat. No. 05-23-010-25MG). An Alzet osmotic pump (DURECT Corporation) was implanted subcutaneously into the mice and Ang II was infused at a rate of 2.5 mg/(kg·day) for 14 days to create a cardiac hypertrophy model^18^. Control mice were implanted subcutaneously with an Alzet osmotic pump containing a solvent. After 14 days, the mice were anaesthetized with 3-4% isoflurane gas and euthanised by cervical dislocation. After being perfused with saline, the hearts were weighed and placed in liquid nitrogen for further analysis^19^.

### 2.2. Library preparation and tRF and tiRNA sequencing

Prior to sequencing, the integrity and quantity of each RNA sample were checked with agarose gel electrophoresis and NanoDrop ND-1000 instruments. tRNA-derived fragments (tRF and tiRNA) are heavily decorated with RNA modifications that interfere with small RNA-seq library construction. Total RNA samples were first pretreated as follows to remove some RNA modifications that interfere with small RNA-seq library construction: 3'-aminoacyl (charged) deacylation to 3'-OH for 3'-adaptor ligation, 3'-cP (2',3'-cyclic phosphate) removal to 3'-OH for 3'-adaptor ligation, 5'-OH (hydroxyl group) phosphorylation to 5'-P for 5'-adaptor ligation, and m1A and m3C demethylation for efficient reverse transcription. The pretreated total RNA was then used to prepare the sequencing library by the following steps: 1) 3' adaptor ligation, 2) 5' adaptor ligation, 3) cDNA synthesis, 4) PCR amplification, and 5) size selection of 134∼160 bp PCR-amplified fragments (corresponding to the 14∼40 nt small RNA size range). Libraries were denatured as single-stranded DNA molecules, captured on Illumina flow cells, amplified in situ as sequencing clusters and sequenced for 50 cycles on the Illumina NextSeq 500 system according to the manufacturer's instructions.

### 2.3. Data analysis of tsRNAs

Cytoplasmic tRNA sequences were downloaded from GtRNAdb. Mitochondrial tRNA sequences were predicted with tRNAscan-SE. Image analysis and base calling were performed with the Solexa pipeline v1.8 (Off-Line base Caller software, v1.8). Sequence quality was checked with FastQC, and the trimmed reads were compared to allow only one mismatch of mature tRNA sequences. The unmapped reads were then compared to allow only one mismatch of precursor tRNA sequences with Bowtie software. The abundance of the tsRNAs was assessed with their sequencing counts normalized to counts per million total aligned reads (CPMs). Differentially expressed tsRNAs were screened with the R package edgeR. Principal component analysis (PCA), correlation analysis, pie charts, Venn diagrams, hierarchical clustering, scatter plots, and volcano plots were generated in the R or Perl environments to statistically calculate and map the expressed tsRNAs^20^.

The tsRFun (https://rna.sysu.edu.cn/tsRFun/index.php)^21^, tRFTar (http://www.rnanut.net/tRFTar/#)^22^, tsRBase (http://tsrbase.org/index.php)^23^, miRDB (https://mirdb.org/mirdb/index.html)^24^, miRanda (http://www.microrna.org)^25^, and TCGA (https://www.cancer.gov/ccg/research/genome-sequencing/tcga) databases were used for tsRNA target gene prediction, pan-cancer analysis, survival analysis, and other bioinformatics analyses.

### 2.4. Isolation and culture of NMVMs

NMVMs were isolated from 1-3-day-old C57BL/6J neonatal mice. The cardiomyocytes were cultured at a density of 7×10⁴ cells/cm² in Petri dishes containing DMEM and M199 medium (Sigma-Aldrich) at a volume ratio of 3:1, supplemented with 5% FBS, 10% horse serum (Sigma-Aldrich), 0.1 mM 5-bromo-2-deoxyuridine (Sigma-Aldrich), and antibiotics (100 U/mL penicillin G and 100 U/mL streptomycin). The cells were cultured at 37 °C in an incubator containing 5% CO₂ and 95% air (v/v)^26^.

### 2.5. Transfection with the tRF-Glu-CTC-013 mimic and inhibitor

The sequences of the negative control (NC), the tRF-Glu-CTC-013 mimic (Mimic), the negative control inhibitor (NCi), and the tRF-Glu-CTC-013 inhibitor (Inhibitor) were as follows (5'-3'): negative control sense: UUCUCCGAACGUGUCACGUTT; negative control antisense: ACGUGACACGUUCGGAGAATT; tRF-Glu-CTC-013 mimic sense: UCCCUGGUGGUCAGUGGUUAGGAUUCGGC; tRF-Glu-CTC-013 mimic antisense sense: CGAAUCCUAACCACUAGACCACCAGGGAUU; inhibitor negative control: CAGUACUUUUGUGUAGUACAA; and tRF-Glu-CTC-013 inhibitor: GCCGAAUCCUAACCACUAGACCACCAGGGA. The NC, Mimic, NCi and Inhibitor were purchased from Sangon Biotech (Shanghai). When the cell density reached 70%, the NC, Mimic, NCi and Inhibitor were transfected into NMVMs with the RNAiMAX transfection reagent (Thermo Fisher, USA). Twenty-four hours after transfection, the medium was replaced with fresh DMEM. The cells were then treated with Ang II for 24 h before being harvested^26^.

### 2.6. Hematoxylin-eosin(H&E) staining, Masson staining, Sirius red staining, and wheat germ agglutinin (WGA) staining

The perfused mouse hearts were excised and fixed in 4% paraformaldehyde before being embedded in paraffin. The hearts were sectioned to a thickness of 5 μm. Cardiomyocyte morphology and inflammatory infiltration were observed using haematoxylin and eosin (H&E) staining (Cat. No. G1120, Solarbio, China). Collagen deposition and myocardial fibrosis were detected using Masson's trichrome and Sirius red staining kits (Cat. No. G1340 and S8060-5, Solarbio, China). The area of the cardiomyocytes was analysed using WGA staining (Cat. No. I3300, Solarbio, China). Images were analysed using ImageJ software to quantify fibrotic and cardiomyocyte areas, as previously reported.

### 2.7. Luciferase assay

The 3′ untranslated regions (3′ UTRs) of the target genes and their mutant variants were synthesized and digested with SacI and XhoI to generate reporter vectors containing microRNA (miRNA) binding sites (Shengong Co., China). The fragment was then inserted into a luciferase reporter vector (Promega, USA), as previously described. For the luciferase assays, the tRF-Glu-CTC-013 mimic was transfected into HEK-293A cells alongside the luciferase reporter vectors containing the *Tas1r3*-3′ UTR and mutant *Tas1r3*-3′ UTR using transfection reagents (Vigofect, Vigorous Biotechnology, China). Forty-eight hours post-transfection, luciferase activity was assessed using a dual-luciferase reporter gene assay system (MeilunBio), following the manufacturer's protocol^26^.

### 2.8. Quantitative real-time PCR

Total RNA was extracted from myocardial tissue and cells using TRIzol reagent (Invitrogen, Carlsbad, CA, USA). An equal amount of total RNA was extracted from each sample using a First Strand cDNA Synthesis Kit (New England Biolabs). Quantitative PCR was performed using the QuantStudio 3 Real-Time PCR System (Applied Biosystems, Thermo Fisher Scientific, Waltham, MA, USA) with reaction mixtures containing SYBR Green (TaKaRa, Japan). The RT-PCR procedure was as follows: SYBR Green levels were measured at the end of each cycle and gene expression was normalized to Gapdh levels. Three replicates of each reaction were performed and analyzed using the 2^-(ΔΔCt)^ method^26^.

We used the stem‒loop method to detect the expression of tsRNAs; their 3' primers were common primers, but the 5' primers were different. Gene expression was normalized to *U6* levels. We list the primer sequences in the table below^27^, ^28^.

### 2.9. Infections with GFP-LC3 adenovirus and GFP-mRFP tandem-tagged LC3 adenovirus for autophagy and autophagic flux assays

The NMVMs were cultured in a 6-well plate for 24 hours. They were then infected with control adenoviruses or GFP-LC3-Adenovirus (GFP-LC3-Adv) or mRFP-GFP-LC3-Adenovirus (mRFP-GFP-LC3-Adv) at an M.O.I. of 10 for a further 24 hours. After infection, the cells were treated with Ang II. The cells were then observed using inverted fluorescence microscopy. The number of RFP and GFP spots in five fields of view was counted, with at least 50 cells counted in each group, as previously reported^29^.

### 2.10. Western blotting assay

The cells were lysed using a lysis buffer that contained both protease and phosphatase inhibitors. The samples were then subjected to SDS-PAGE and transferred to PVDF membranes. These membranes were then blocked with 5% skimmed milk, incubated with the primary antibodies overnight at 4 °C, washed with TBST, incubated with the secondary antibodies and detected using an enhanced chemiluminescence reagent. TAS1R3 expression, as well as the expression of phosphorylated and total mTOR and LC3B I and II, was detected, with GAPDH used as a protein loading control. Finally, densitometric analysis was performed using ImageJ software^30^.

### 2.11. Statistical analysis

Data analysis was performed with GraphPad Prism 8.0. All the data are expressed as the means ± SEMs. Differences between two groups were analyzed with Student's t test, and differences between multiple groups were analyzed with one-way ANOVA with a Bonferroni correction. Differences between groups were considered significant at *P* < 0.05.

## 3. Results

### 3.1. Establishment of an Ang II-induced myocardial hypertrophy model in mice

**Figure 1:**
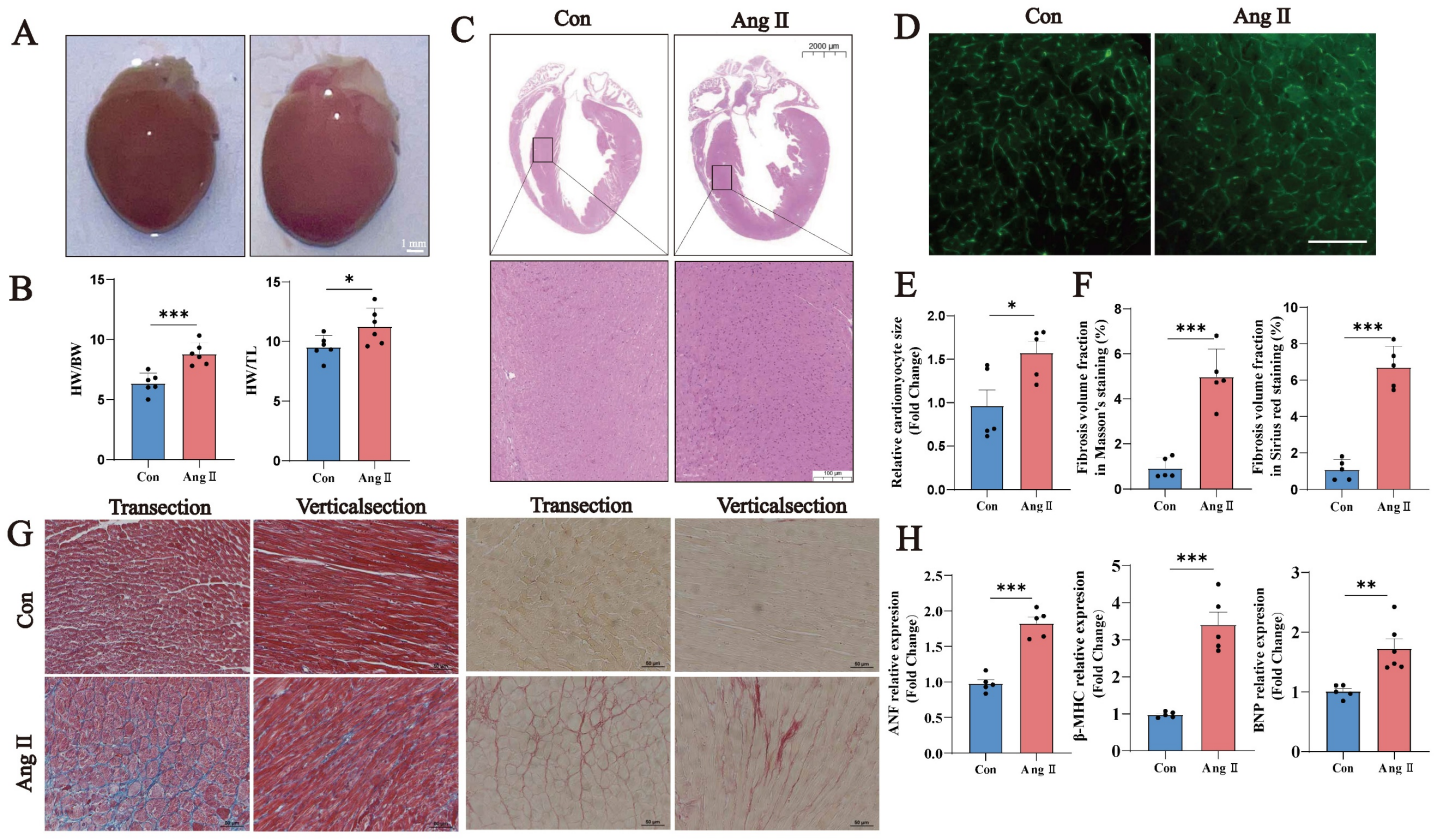
Ang II-induced myocardial hypertrophy mouse model.** (A)** Representative images of hearts from the Con and Ang II groups of mice. Scale bar: 0.5 cm. **(B)** Heart/weight ratios and heart/tibia length ratios of Con- and Ang II-treated mice (n = 6).** (C)** Representative images of H&E staining, scale bar: 2 mm (n = 5).** (D)** Representative images of WGA staining, scale bar: 10 µm (n = 5).** (E)** Semiquantitative analysis of WGA staining (n = 5).** (F)** Semiquantitative analysis of the fibrotic area ratio based on Masson's trichrome and Sirius red staining (n = 5). Left: Masson's staining; right: Sirius red staining. **(G)** Representative images of Masson's trichrome and Sirius red staining, scale bar: 10 µm (n = 5).** (H)** q-PCR was performed to detect *Nppa*, *Nppb* and *Myh7* in myocardial samples from each group. *U6* was used as an internal control. ns: not significant, *P < 0.05, **P < 0.01, ***P < 0.001 vs. the control group.

We constructed a model of Ang II-induced myocardial hypertrophy in mice. In this model, the heart volume, heart weight/body weight ratio, and heart weight/tibia length ratio were increased significantly in Ang II-induced mice (Fig. 1A, B). H&E staining revealed thickened ventricular walls and smaller ventricular chambers in Group A (Fig. 1C). WGA staining revealed an increased cardiomyocyte cross-sectional area and cardiomyocyte hypertrophy (Fig. 1D, E). Masson's trichrome and Sirius red staining revealed a significant increase in the fibrotic area ratio (Fig. 1F, G). The Ang II-induced cardiac hypertrophy markers *Nppa*, *Nppb*, and *Myh7* were significantly increased in the Ang II group (Fig. 1H). These results suggest that Ang II successfully induced a model of cardiac hypertrophy in this study.

### 3.2. Distribution and enrichment analysis of the tsRNAs

To identify the tsRNAs produced in mouse hearts during cardiac hypertrophy, we conducted tsRNA transcriptome sequencing. Many genes are expressed and regulated similarly in cardiac hypertrophy and in the neonatal mouse heart. Therefore, to explore tsRNAs that are more relevant during cardiac hypertrophy, we also examined the tsRNA transcriptome of neonatal mice. There were three mice in each group.

**Figure 2:**
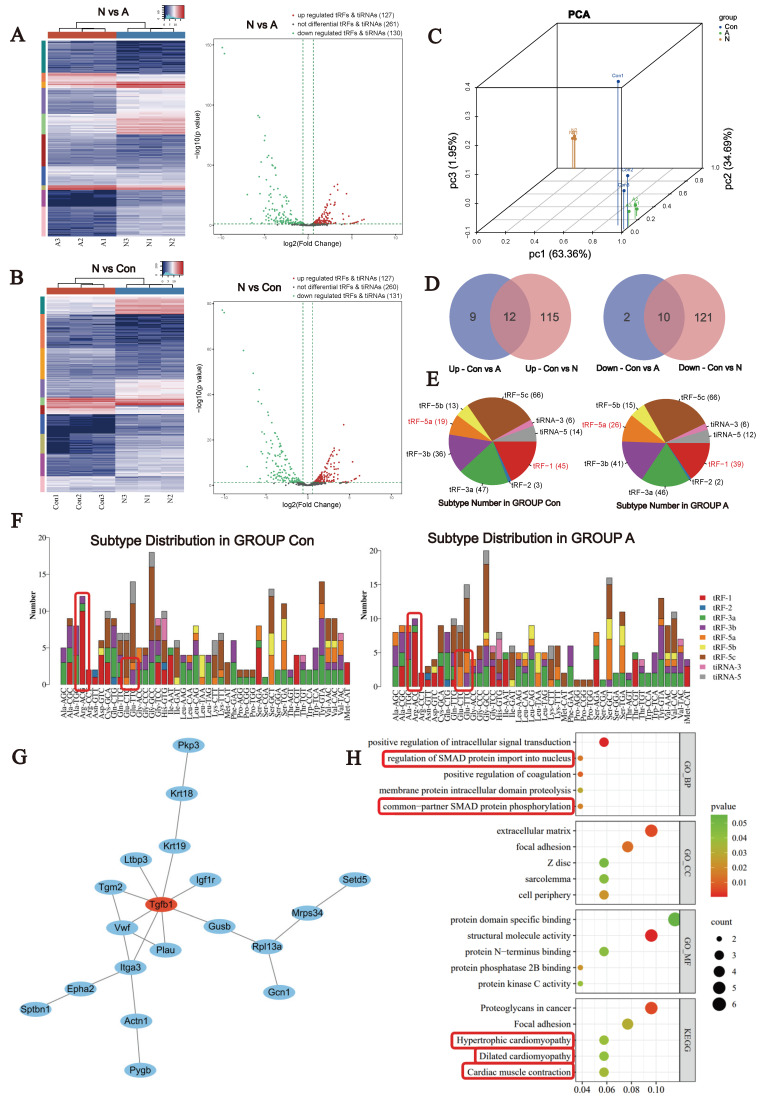
Differential expression of tsRNAs in Ang II-induced myocardial hypertrophy mice.** (A)** Hierarchical clustering analysis and volcano plot of the differentially expressed tsRNAs in the Con and Ang II groups, n = 3.** (B)** Hierarchical clustering analysis and volcano plot of the differentially expressed tsRNAs in the Con and N groups, n = 3.** (C)** Principal component analysis. **(D)** Venn diagram based on the differential up- or downregulation of tsRNAs. Left: upregulated tsRNAs; Right: downregulated tsRNAs**. (E)** Pie chart of the distribution of tRF and tiRNA subtypes.** (F)** The number of tRF and tiRNA subtypes compared with that of tRNA isodecoders. **(G)** The most tightly clustered subnetworks were identified by the MCODE plugin, which was used to identify network gene clustering. The red nodes are the key tissue-specific genes screened. **(H)** Enrichment analyses of the predicted target genes.

A total of 518 tsRNAs were identified in cardiac tissue, many of which were found to exhibit differential expression. Compared with the Con group, 21 tsRNAs were found to be upregulated and 12 downregulated in the A group. In the N group, 126 tsRNAs were found to be upregulated and 131 downregulated compared with the control group (Fig. 2A-C). The Venn diagram (Fig. 2D) revealed 12 upregulated and 10 downregulated tsRNAs in both the AngII-treated and neonatal heart groups compared with the control group. The pie chart shows the distribution of the number of different tsRNAs with a CPM ≥20. The most pronounced responses to Ang II stimulation were a decrease in tRF-1 and an increase in tRF-5a (Fig. 2E). The most significant changes were observed in Arg-ACG- and Glu-CTC/GTC-derived tsRNAs (Fig. 2F). We predicted the downstream target genes of the differentially expressed tsRNAs using a bioinformatics website. Enrichment and protein interaction analyses revealed that the TGF-β1/SMAD signaling pathway was highly enriched. These findings suggest that tsRNAs play an important role in the pathological process of myofibrosis during cardiac hypertrophy. Additionally, signaling pathways associated with hypertrophic cardiomyopathy, dilated cardiomyopathy and myocardial contraction was also enriched (Fig. 2G, H). Taken together, these signaling pathways imply that tsRNAs may have biological functions in cardiac hypertrophy, myocardial remodeling, and energy metabolism.

### 3.3. The expression of tsRNAs increased in the Ang II-induced NMVM hypertrophy model

**Figure 3:**
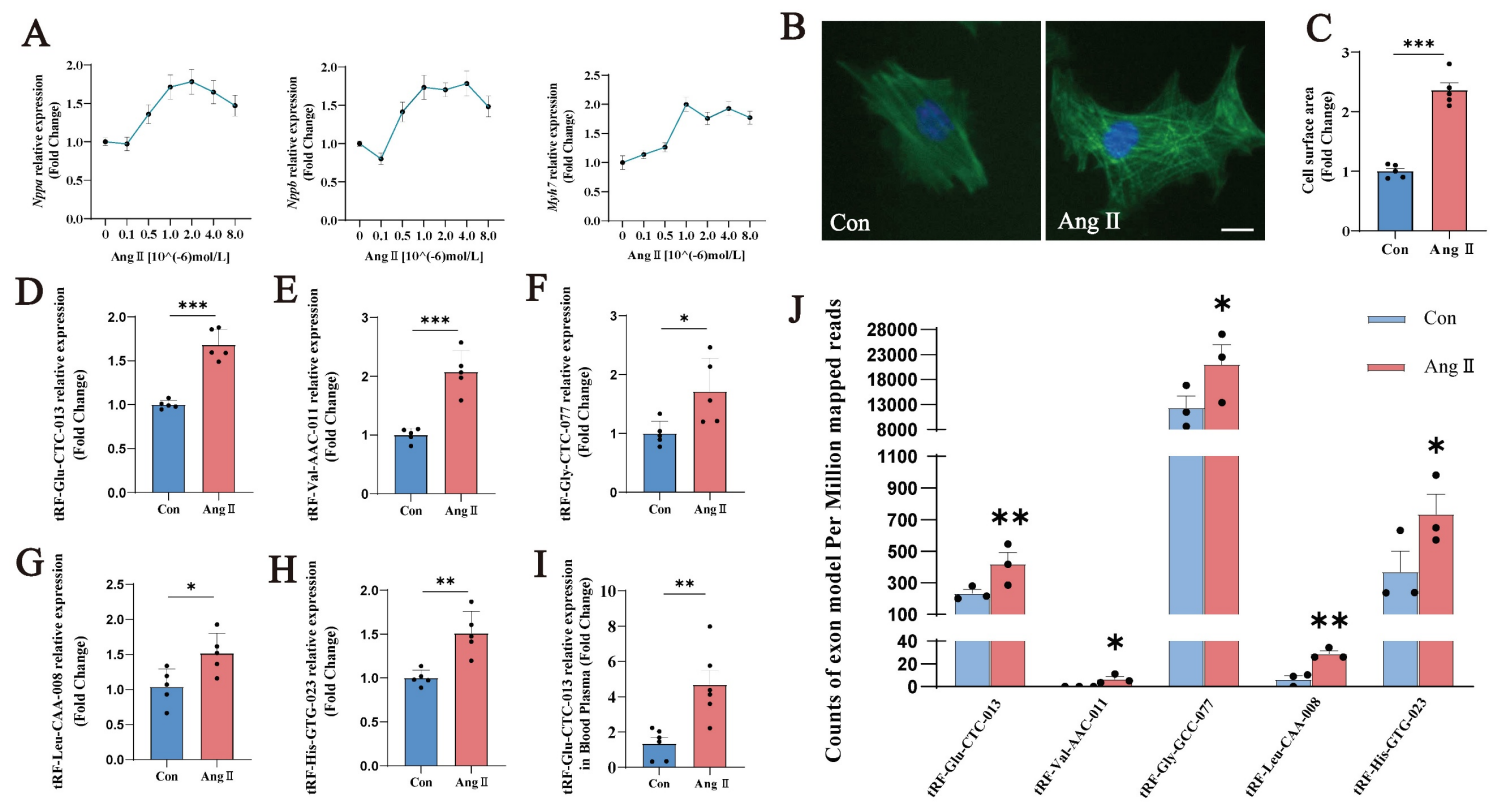
Validation of differentially expressed tsRNAs by modeling Ang II-induced NMVMs. **(A)** Changes in the levels of cardiac hypertrophy markers in the NMVM model induced by different concentrations of Ang II. Units: 10^-6^ mol/L. **(B, C)** Images of the cell area labeled with Actin-Tracker Green staining and quantitative analysis of all groups (n = 5). **(D-H)** q-PCR was performed to detect tRF-Glu-CTC-013, tRF-Val-AAC-011, tRF-Gly-GCC-077, tRF-Leu-CAA-008, and tRF-His-GTG-023 in NMVMs from each group. (n = 5). **(I)** Expression of tRF-Glu-CTC-013 in the plasma of the Con and Ang II groups (n = 6). **(J)** The number of exon models per million mapped reads of tRF-Glu-CTC-013, tRF-Val-AAC-011, tRF-Gly-GCC-077, tRF-Leu-CAA-008, and tRF-His-GTG-023 (n = 3). *U6* was used as an internal control. ns: not significant, *P < 0.05, **P < 0.01, ***P < 0.001 vs. control group.

To investigate and validate the observed changes in tsRNAs in cardiomyocytes further, we created an Ang II-induced NMVMs model and examined the expression of relevant tsRNAs. Different doses of Ang II were used to induce the NMVMs, and the mRNA expression of *Nppa*, *Nppb*, and *Myh7* was assessed, as was the cell surface area. A concentration of 10⁻⁶ mol/L was selected for subsequent experiments (Fig. 3A-C).

As many pathological processes of cardiac hypertrophy are mechanistically similar to developmental processes in the neonatal heart, our study focused on simultaneous differential tsRNA expression in neonatal mice with and without cardiac hypertrophy. Previous studies have shown that tsRNA expression increases significantly during cardiac hypertrophy development due to increased Ang II and tRNA cleavage. Therefore, we validated the simultaneous increase in tsRNAs in Groups A and N.

In the Ang II-induced myocardial hypertrophy model in NMVMs, the expression of tRF-Glu-CTC-013, tRF-Val-AAC-011, tRF-Gly-GCC-077, tRF-Leu-CAA-008 and tRF-His-GTG-023 was significantly increased (Fig. 3D-H; Table 3). Furthermore, the exon model counts per million mapped reads for tRF-Glu-CTC-013, tRF-Val-AAC-011, tRF-Gly-GCC-077, tRF-Leu-CAA-008 and tRF-His-GTG-023 differed significantly. With the exception of tRF-Val-AAC-011 and tRF-Leu-CAA-008, the remaining three tsRNAs exhibited relatively high expression levels. Furthermore, we examined the expression of tRF-Glu-CTC-013 in mouse plasma and found that it was significantly increased in the Ang II group. This suggests that tRF-Glu-CTC-013 could be used as a marker of cardiac hypertrophy (Fig. 3I). tRF-Glu-CTC-013 showed the most significant differential expression and was highly upregulated (Fig. 3J).

### 3.4. tRF-Glu-CTC-013 attenuated myocardial hypertrophy by reducing inflammation and fibrosis

**Figure 4:**
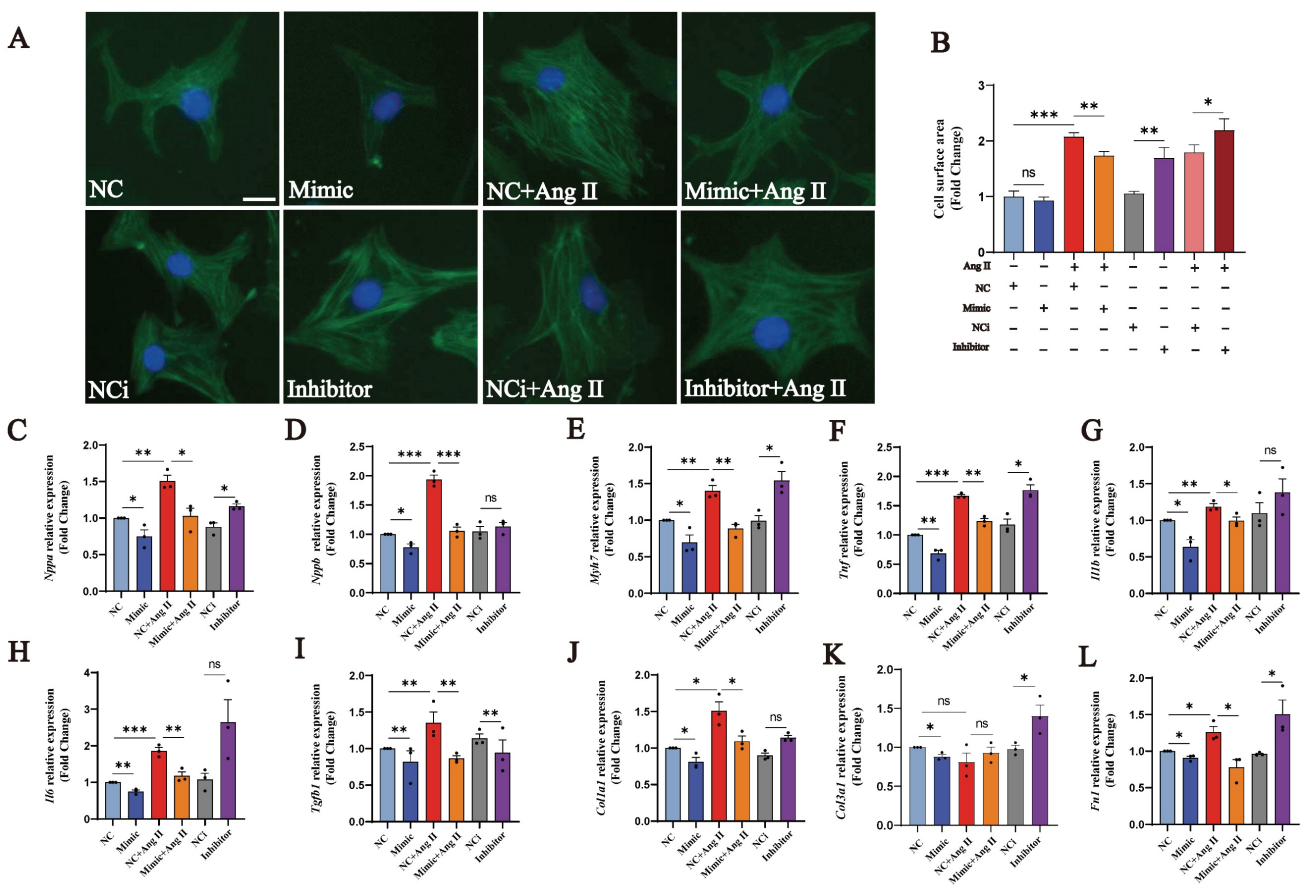
tRF-Glu-CTC-013 counteracts myocardial hypertrophy by reducing myocardial inflammation and fibrosis. **(A, B)** Images of the cell area labeled with Actin-Tracker Green and quantitative analysis of the NC, Mimic, NC+Ang II, Mimic + Ang II, NCi, Inhibitor, NCi+Ang II, and Inhibitor+Ang II groups (n = 3). **(C-L)** q-PCR was performed to assess *Nppa*, *Nppb*, *Myh7*, *Tnf*, *Il1b*, *Il6*, *Tgfb1*, *Col1a1*, *Col3a1*, and *Fn1* in the NMVMs from each group. *U6* was used as an internal control (n = 3). ns: not significant, *P < 0.05, **P < 0.01, ***P < 0.001 vs. the control group.

In order to investigate whether tRF-Glu-CTC-013 has a biological function, we synthesised mimics and inhibitors of tRF-Glu-CTC-013, which were then used to transfect NMVMs in order to explore its biological role. We found that tRF-Glu-CTC-013 significantly reduced the Ang II-induced increase in cardiomyocyte surface area, as well as reducing the expression of the cardiac hypertrophy markers *Nppa*, *Nppb* and *Myh7* (Fig. 4A-E). An increase in inflammatory factors is an essential part of cardiac hypertrophy. Therefore, we also investigated whether tRF-Glu-CTC-013 could reduce inflammation during cardiac hypertrophy development. We found that the tRF-Glu-CTC-013 mimic reduced the expression of *Tnf*, *Il1b*, and *Il6*, exhibiting an anti-inflammatory effect in the NMVM hypertrophy model (Fig. 4F-H). The TGF-β/SMAD signalling pathway is activated during cardiac hypertrophy development, promoting the production of cellular collagen and the extracellular matrix and ultimately leading to myocardial fibrosis. We found that tRF-Glu-CTC-013 reduced the expression of *Tgfb1*, *Col1a1*, *Col3a1* and *Fn1*, thereby inhibiting myocardial fibrosis (Fig. 4I-L).

### 3.5. Predicting the target genes of tRF-Glu-CTC-013 and analyzing its biological function

**Figure 5:**
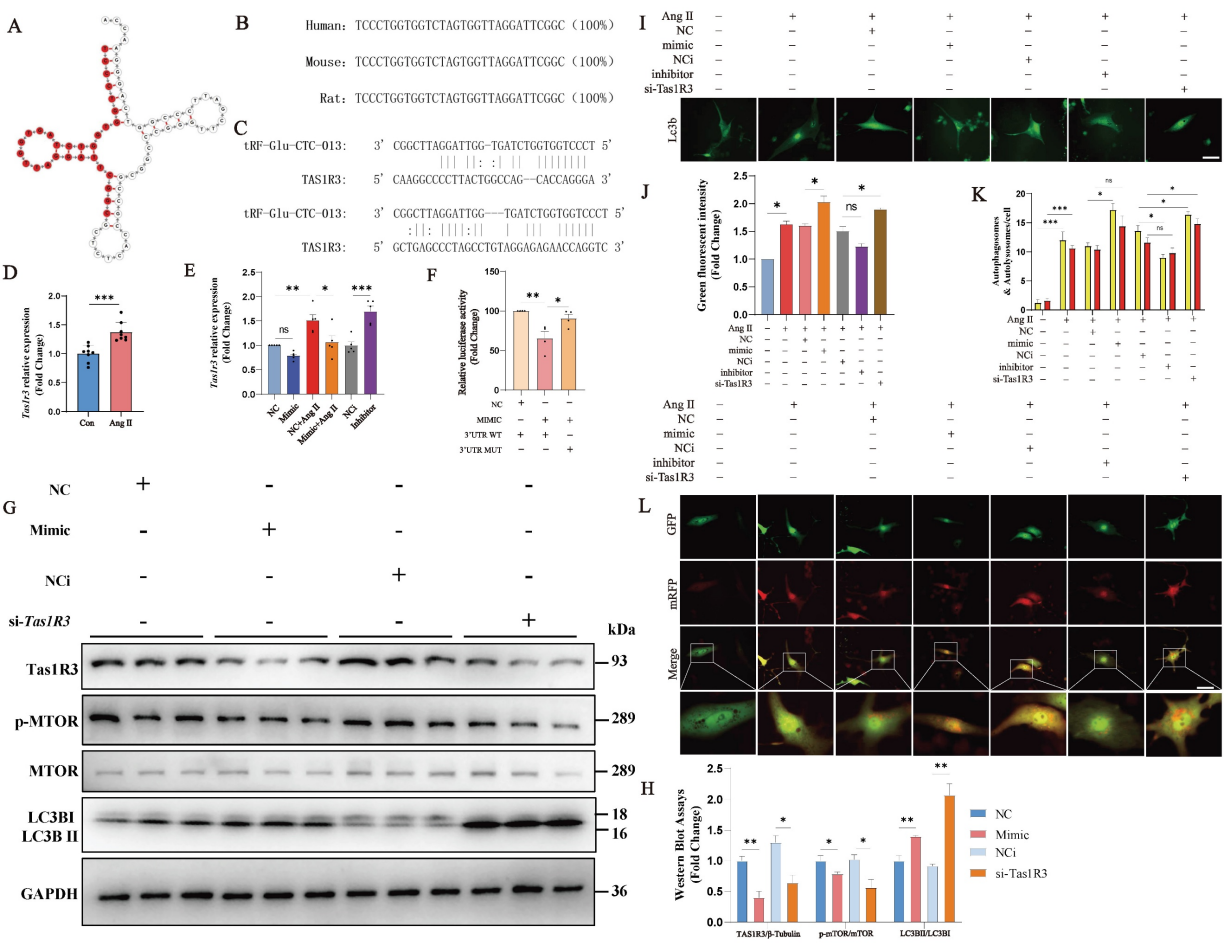
tRF-Glu-CTC-013 increases autophagy in NMVMs by direct inhibition of TAS1R3. **(A)** Schematic representation of the tRNA from which tRF-Glu-CTC-013 is derived.** (B)** Human, mouse and rat sequences of tRF-Glu-CTC-013. **(C)** Binding site of tRF-Glu-CTC-013 in the 3'UTR of *Tas1r3* predicted by the miRDB and miRanda databases.** (D, E)** q-PCR was performed to assess *Tas1r3* in the NMVMs from each group. *Gapdh* was used as a loading control (n = 8). **(F)** The repressive effect of tRF-Glu-CTC-013 on the activity of the 3′UTR was measured by a luciferase assay (n = 4). **(G, H)** The expression levels of TAS1R3, phosphorylated and total mTOR, and LC3B I and II were determined by immunoblotting in NMVMs subjected to different treatments (n = 3). GAPDH was used as a loading control. **(I, J)** NMVMs were transfected with NC, the tRF-Glu-CTC-013 mimic, NCi, the tRF-Glu-CTC-013 inhibitor, or *Tas1R3* siRNA (si-*Tas1R3*) for 48 h and then infected at an M.O.I. of 10 with GFP-LC3 adenovirus (GFP-LC3-Adv) and treated with Ang II for 24 h to analyze the number of autophagic vesicles (n = 5). Scale bar: 50 µm.** (K, L)** NMVMs were transfected with NC, the tRF-Glu-CTC-013 mimic, NCi, the tRF-Glu-CTC-013 inhibitor, or si-*Tas1R3* for 48 h and then infected at an M.O.I. of 10 with tandem fluorescence-labeled LC3 adenovirus (mRFP-GFP-LC3-Adv) and treated with Ang II for 24 h to detect autophagic flux. The red spots represent the number of intracellular autolysosomes. The yellow spots indicate the number of intracellular autophagosomes (n = 5). Scale bar: 50 µm. ns, not significant; *P < 0.05, **P < 0.01, ***P < 0.001 vs. the control group.

tRF-Glu-CTC-013 is a small RNA fragment located at the 5' end of tRNA-Glu which is cleaved off. Using a bioinformatics website, we established that the tRF-Glu-CTC-013 sequence is highly conserved in humans, rats and mice, and that its predicted target genes are the most biologically significant (Fig. 5A, B).

In order to predict the molecular mechanisms associated with tRF-Glu-CTC-013, we used the miRDB and miRanda websites to predict the downstream targets of tRF-Glu-CTC-013 in a microRNA-like mode of action, and then validated the predicted targets using NMVMs. We found that the target gene* Tas1r3* was negatively regulated by tRF-Glu-CTC-013. Ang II was found to induce *Tas1r3* expression in an Ang II-induced NMVMs model (Fig. 5C, D). tRF-Glu-CTC-013 was found to inhibit Tas1r3 mRNA expression, and conversely, blocking tRF-Glu-CTC-013 resulted in increased *Tas1r3* mRNA expression (Fig. 5E). A luciferase assay revealed that tRF-Glu-CTC-013 bound well to the 3' UTR of Tas1r3 (Fig. 5F). Tas1r3 expression was inhibited by tRF-Glu-CTC-013, which in turn prevented the phosphorylation of mTOR, leading to a significant increase in LC3B II/I. These findings suggest that tRF-Glu-CTC-013 increases autophagy by inhibiting TAS1R3, which benefits cardiomyocytes under pathological conditions (Fig. 5G, H).

To investigate whether tRF-Glu-CTC-013 affects the autophagy function of cardiomyocytes via the TAS1R3 pathway, the cells were transfected with tRF-Glu-CTC-013 mimics. The cells were then treated with Ang II for 24 hours after being transfected with either the tRF-Glu-CTC-013 inhibitor or *Tas1r3* siRNA for 48 hours, and then with GFP-LC3 adenovirus (GFP-LC3-Adv) or GFP- and mRFP tandemly tagged LC3 adenovirus (mRFP-GFP-LC3-Adv). Autophagy regulation in the cardiomyocytes was investigated by analyzing the number of autophagic vesicles, autophagosomes, and lysosomes that formed autophagolysosomes (Fig. 5I-L). Transfection with the tRF-Glu-CTC-013 mimic promoted the formation of autophagic vesicles and autophagic flux in cardiomyocytes, whereas transfection with the tRF-Glu-CTC-013 inhibitor reduced the formation of autophagic vesicles and autophagic flux. Autophagic vesicle formation and autophagic flux were also significantly increased by *Tas1r3* siRNA-mediated knockdown.

## 4. Discussion

tsRNAs are a newly discovered class of regulatory non-coding RNAs that are emerging as important factors in cardiac pathophysiology 31. Age-related cardiac remodeling, characterized by cardiomyocyte senescence, fibrotic deposition, and functional decline, is mechanistically linked to oxidative stress, mitochondrial dysfunction, and autophagic imbalance—processes modulated by both renin‒angiotensin system (RAS) signaling and non-coding RNA networks^2^, ^32^, ^33^. Pathological activation of the RAS, particularly Ang II overproduction, creates a molecular environment conducive to tsRNA dysregulation, which can exacerbate cardiac dysfunction^32^, ^34^. Building on this concept, our study identifies Ang II-induced tsRNA alterations as a key mediator of hypertrophic remodeling. Notably, tRF-Glu-CTC-013 plays a multifaceted protective role by inhibiting inflammation, fibrosis and cardiomyocyte hypertrophy.

Through comprehensive tsRNA profiling in an Ang II-challenged mouse model, we identified 33 differentially expressed tsRNAs (21 of which were upregulated and 12 of which were downregulated). Previous studies have demonstrated that tsRNA expression increases significantly under stressful conditions. Therefore, the expression of the 21 upregulated tsRNAs may have greater biological significance. Of the five validated candidates (tRF-Glu-CTC-013, tRF-Val-AAC-011, tRF-Gly-GCC-077, tRF-Leu-CAA-008, and tRF-His-GTG-023), tRF-Glu-CTC-013 exhibited exceptional stability and evolutionary conservation, as well as extensive interaction with target genes, suggesting the need for further functional analysis. In NMVMs, overexpression of tRF-Glu-CTC-013 attenuated Ang II-induced cellular hypertrophy while suppressing inflammation, fibrosis, and pathological remodeling.

A previous study revealed a correlation between cardiac hypertrophy and tsRNAs, and demonstrated for the first time that tRFs respond to the effects of cardiac hypertrophy stress^31^. The expression of tRFs increased significantly in ISO-induced cardiac hypertrophy in rats. Furthermore, tRFs1 and tRFs2 were found to increase the surface area of H9c2 cells and the expression of genes associated with cardiac hypertrophy^13^. Shen investigated the relationship between tsRNAs and cardiac hypertrophy, reporting that altered tsRNAs in individuals with cardiac hypertrophy could lead to intergenerational inheritance. Compared with this study, our research has several advantages. Firstly, we used Ang II to create a more accurate model of cardiac hypertrophy in pathological states and analyzed changes in tsRNA expression. Secondly, in contrast to Shen's study, we validated tsRNAs using the Ang II-induced cardiac hypertrophy cell model of NMVMs. This enabled us to realistically screen for tsRNAs that change at the onset of cardiac hypertrophy.

**Figure 6:**
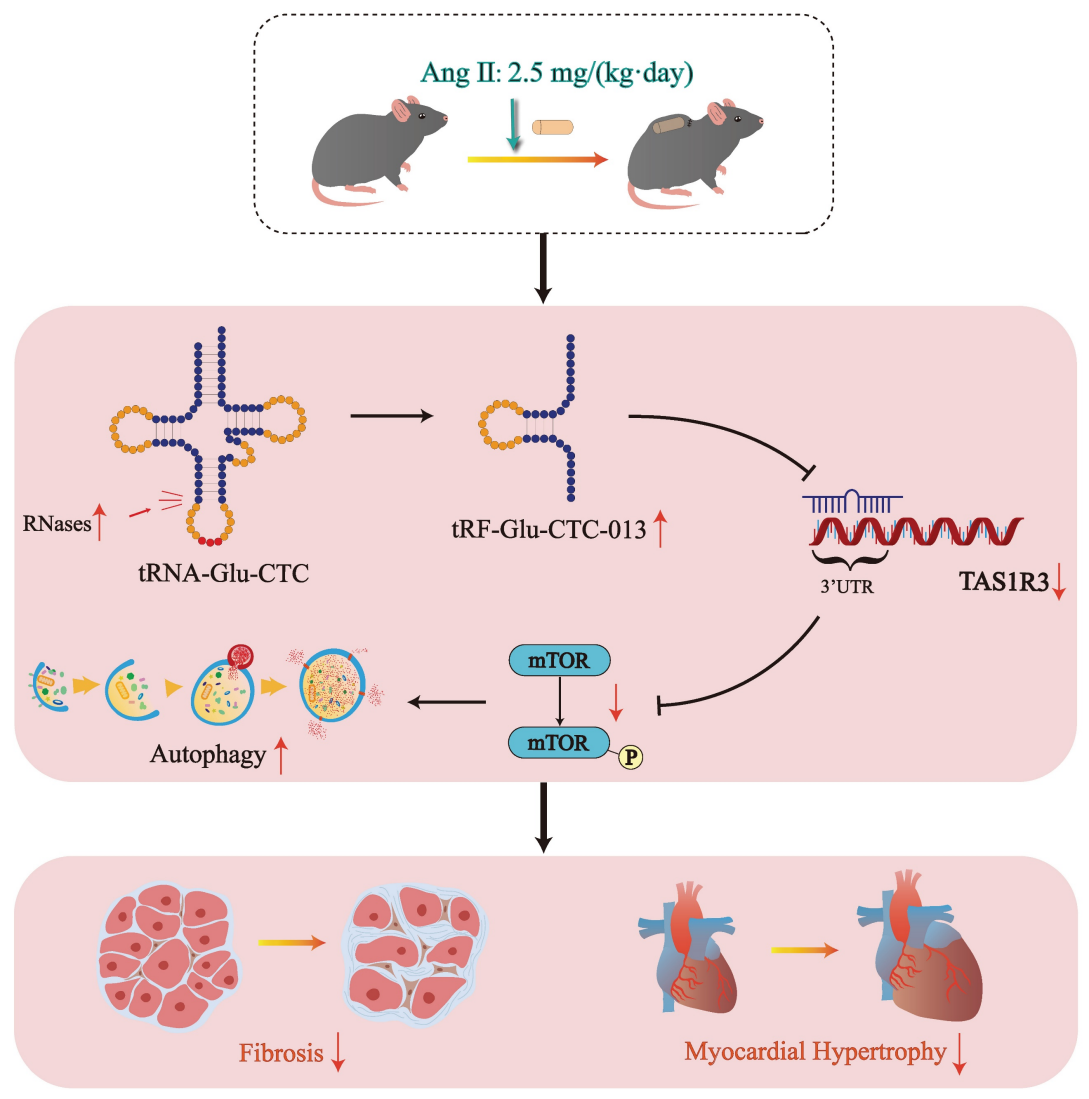
Schematic diagram of the generation of tRF-Glu-CTC-013 and its cardioprotective effects.

In this study, we discovered that tRF-Glu-CTC-013 binds specifically to the 3' UTR of TAS1R3, thereby inhibiting its expression. TAS1R3, which is also known as the sweet taste receptor, is an amino acid receptor found in taste neurons^35^. Not only does TAS1R3 play a role in sweet taste neurons, it also acts as a glucose sensor in the gut and hypothalamus^36^, ^37^. TAS1R3 has been shown to inactivate mTORC1 alongside TAS1R1 in order to maintain important physiological functions. Wauson et al. reported that S6 phosphorylation of mTOR in the heart was reduced by 60% in *Tas1r3*^ (-/-)^ mice. Knockdown of *Tas1r3* in the H9c2 cell line resulted in a significant decrease in mTORC1 phosphorylation, inducing an increase in autophagy^38^. However, increased TAS1R3 in a physiological state may lead to impaired autophagic flux. In cardiac hypertrophy, though, increased autophagic flux is beneficial for clearing injured and dysfunctional cells from cardiac tissue^38^. Therefore, in myocardial hypertrophy, tRF-Glu-CTC-013 targets and inhibits *Tas1r3*, thereby promoting autophagy and attenuating inflammation, myocardial hypertrophy and fibrosis (Fig. 6).

One limitation of this study is that the biological function of tRF-Glu-CTC-013 was not confirmed through animal experimentation. Furthermore, while we showed that tRF-Glu-CTC-013 is a promising biomarker for diagnosing cardiac hypertrophy in mice, further evaluation of blood samples from patients with cardiac hypertrophy is required to confirm its potential as a serum marker.

In the future, it will be possible to develop tRF-Glu-CTC-013 mimics as a therapeutic drug to treat cardiac hypertrophy, attenuating myocardial remodeling and slowing or inhibiting the pathological process in affected patients. However, due to the one-to-many nature of non-coding small RNAs, it is necessary to further refine the molecular mechanism and biological function of tRF-Glu-CTC-013 in order to avoid off-target or adverse clinical effects. Our study demonstrates the powerful biological functions of tsRNAs and highlights their importance in the future clinical treatment of cardiac hypertrophic diseases.

## 5. Conclusions

This study found that various tRFs were enriched in hearts with cardiac hypertrophy, with tRF-Glu-CTC-013 acting as a biological marker and exhibiting functions that reduce inflammation, myocardial hypertrophy, and fibrosis. Furthermore, tRF-Glu-CTC-013 was found to target *Tas1r3*, thereby inhibiting its expression and enhancing autophagy. These results may improve our understanding of the pathogenesis of cardiac hypertrophy and inform the development of new therapeutic strategies.

## Figures and Tables

**Table 1:** Primer sequences for RT-PCR

Primer	Sequences (5′-3′)
*Nppa* (*Anf*) forward	GTGCGGTGTCCAACACAGAT
*Nppa* (*Anf*) reverse	TCCAATCCTGTCAATCCTACCC
*Nppb* (*Bnp*) forward	AATTCAAGATGCAGCTGCTG
*Nppb* (*Bnp*) reverse	GAATTTTGAGGTCTCTGCTGG
*Myh7* (*β-MHC*) forward	ACTGTCAACACTAAGAGGGTCA
*Myh7* (*β-MHC*) reverse	TTGGATGATTTGATCTTCCAGGG
*Tnf* (*Tnf-α*) forward	ACGGCATGGATCTCAAAGAC
*Tnf* (*Tnf-α*) reverse	GTGGGTGAGGAGCACGTAGT
*Il1b* (*Il-1β*) forward	CAGGCAGGCAGTATCACTCA
*Il1b* (*Il-1β*) reverse	TGTCCTCATCCTGGAAGGTC
*Il6* (*Il-6*) forward	CCGGAGAGGAGACTTCACAG
*Il6* (*Il-6*) reverse	TCCACGATTTCCCAGAGAGAAC
*Tgfb1* (*Tgf-β*) forward	CTTCAATACGTCAGACATTCGGG
*Tgfb1* (*Tgf-β*) reverse	GTAACGCCAGGAATTGTTGCTA
*Col1a1* forward	TGACTGGAAGAGAGCGGAGAGT
*Col1a1* reverse	GACGGCTGAGTAGGGAACAC
*Col3a1* forward	CGTAAGCACTGGTGGACAGA
*Col3a1* reverse	AGCTGCACATCAACGACATC
*Fn1* forward	TTCAAGTGTGATCCCCATGAAG
*Fn1* reverse	CAGGTCTACGGCAGTTGTCA
*Tas1r3* forward	GGTGCCCTACTGCCTGAATT
*Tas1r3* reverse	TGCCCCATCACATGTTCCTC
*Gapdh* forward	AACTTTGGCATTGTGGAAGG
*Gapdh* reverse	ACACATTGGGGGTAGGAACA

**Table 2:** Primer sequences for tsRNAs

tsRNA primer	Sequences (5′-3′)
tRF-Glu-CTC-013 PCR	TCCCTGGTGGTCTAGTGGTTAGGATTCGGC
tRF-Glu-CTC-013 RT	GTCGTATCCAGTGCAGGGTCCGAGGTATTCGCACTGGATACGACGCCGAATC
tRF-Val-AAC-011 PCR	GTTTCCGTAGTGTAGTGGTCATCACGCTCGC
tRF-Val-AAC-011 RT	GTCGTATCCAGTGCAGGGTCCGAGGTATTCGCACTGGATACGACGCGAGCG
tRF-Gly-GCC-077 PCR	GCATTGGTGGTTCAGTGGTAGAATTCTCGCC
tRF-Gly-GCC-077 RT	GTCGTATCCAGTGCAGGGTCCGAGGTATTCGCACTGGATACGACGGCGAG
tRF-Leu-CAA-008 PCR	GTCAGGATGGCCGAGTGGTCTAAG
tRF-Leu-CAA-008 RT	GTCGTATCCAGTGCAGGGTCCGAGGTATTCGCACTGGATACGACCTTAGAC
tRF-His-GTG-023 PCR	GCCGTGATCGTATAGTGGTTAGTACTCTGCG
tRF-His-GTG-023 RT	GTCGTATCCAGTGCAGGGTCCGAGGTATTCGCACTGGATACGACCGCAGAG
*U6* PCR	GCGCGTCGTGAAGCGTTC
*U6* RT	GTCGTATCCAGTGCAGGGTCCGAGGTATTCGCACTGGATACGACAAAATATG
Universal Primer	GTGCAGGGTCCGAGGT

**Table 3:** List of validated tsRNAs

Name	Sequence	Type	Fold Change	*P* value
tRF-Glu-CTC-013	TCCCTGGTGGTCTAGTGGTTAGGATTCGGC	tRF-5c	1.840170303	0.007167246
tRF-Val-AAC-011	GTTTCCGTAGTGTAGTGGTCATCACGCTCGC	tRF-5c	16.83846362	0.048665212
tRF-Gly-GCC-077	GCATTGGTGGTTCAGTGGTAGAATTCTCGCC	tRF-5c	1.758395242	0.016178725
tRF-Leu-CAA-008	GTCAGGATGGCCGAGTGGTCTAAG	tRF-5b	3.664965098	0.003331476
tRF-His-GTG-023	GCCGTGATCGTATAGTGGTTAGTACTCTGCG	tRF-5c	2.007033479	0.011403364

## Abbreviations

tsRNA: tRNA-derived small RNA
Ang II: angiotensin II
tiRNA: tRNA-derived stress-induced RNA
tRF: tRNA-derived fragment
TIMP3: tissue inhibitor of metalloproteinases3
ISO: isoprenaline
PCA: principal component analysis
NMCMs: neonatal mouse ventricular myocytes
NC: negative control
MIMIC: tRF-Glu-CTC-013 mimic
NCi: negative control inhibitor
INHIBITOR: tRF-Glu-CTC-013 inhibitor
WGA: wheat germ agglutinin
ANF(NPPA): natriuretic peptide A
BNP: *natriuretic peptides A-like*
β-MHC(Myh7): myosin, heavy polypeptide 7, cardiac muscle, beta
TGF-β1: *transforming growth factor beta 1*
SMAD: *small mothers against decapentaplegic*
COL1A1: collagen type I alpha 1 chain
COL3A1: collagen type III alpha 1 chain
FN1: fibronectin 1
TNF-α: tumor necrosis factor alpha-like
IL-1β: interleukin 1 beta
IL-6: interleukin 6
GAPDH: glyceraldehyde-3-phosphate dehydrogenase
TAS1R3: taste 1 receptor member 3
LC3B(MAP1LC3B): microtubule associated protein 1 light chain 3 beta
mTOR: mechanistic target of rapamycin kinase
ROS: reactive oxygen species
